# Overexpression of miR-200s inhibits proliferation and invasion while increasing apoptosis in murine ovarian cancer cells

**DOI:** 10.1371/journal.pone.0307178

**Published:** 2024-07-19

**Authors:** Resh Carter, Jim J. Petrik, Roger A. Moorehead

**Affiliations:** Department of Biomedical Sciences, Ontario Veterinary College, University of Guelph, Guelph, ON, Canada; Chung Shan Medical University, TAIWAN

## Abstract

Women diagnosed with ovarian cancer frequently have a poor prognosis as their cancer is often diagnosed at more advanced stages when the cancer has metastasized. At this point surgery cannot remove all the tumor cells and while ovarian cancer cells often initially respond to chemotherapeutic agents like carboplatin and paclitaxel, resistance to these agents frequently occurs. Thus, novel therapies are required for the treatment of advanced stage ovarian cancer. One therapeutic option being explored is the regulation of non-coding RNAs such as microRNAs. An advantage of microRNAs is that they can regulate tens, hundreds and sometimes thousands of mRNAs in cells and thus may be more effective than chemotherapeutic agents or targeted therapies. To investigate the therapeutic potential of miR-200s in ovarian cancer, lentiviral vectors were used to overexpress both miR-200 clusters in two murine ovarian cancer cell lines, ID8 and 28–2. Overexpression of miR-200s reduced the expression of several mesenchymal genes and proteins, significantly inhibited proliferation as assessed by BrdU flow cytometry and significantly reduced invasion through Matrigel coated transwell inserts in both cell lines. Overexpression of miR-200s also increased basal apoptosis approximately 3-fold in both cell lines as determined by annexin V flow cytometry. Pathway analysis of RNA sequencing of control and miR-200 overexpressing ovarian cancer cells revealed that genes regulated by miR-200s were involved in processes like epithelial mesenchymal transition (EMT) and cell migration. Therefore, miR-200s can inhibit proliferation and increase apoptosis while suppressing tumor cell invasion and thus simultaneously target three key cancer pathways.

## Introduction

Globally, ovarian cancer is the most lethal gynecological cancer with over 300,000 cases and over 200,000 deaths in 2020 [1]. There are 5 main ovarian cancer subtypes, high grade serous ovarian cancer (HGSOC), endometrioid carcinoma (EC), clear cell carcinoma (CCC), mucinous carcinoma (MC) and low grade serous ovarian cancer (LGSOC) with HGSOC being the most prevalent form [2]. The lack of specific symptoms associated with ovarian cancer (i.e. bloating, pain, nausea) means that ovarian cancer is typically diagnosed at later disease stages where the five year survival rate is approximately 29% [3]. There are several risk factors including an increased number of ovulatory cycles, endometriosis, polycystic ovarian syndrome, and genetic factors like *BRCA1*/*BRCA2* mutations [3]. Treatment of ovarian cancer typically involves debulking surgery followed by chemotherapy with platinum-based chemotherapies like cisplatin or carboplatin in combination with paclitaxel [4]. For ovarian cancer patients with defects in homologous DNA repair, such as those with *BRCA1* or *BRCA2* mutations, Poly (ADP-ribose) polymerase (PARP) inhibitors like olaparib and niraparib have shown survival benefits in several clinical trials [5–9]. As most ovarian cancers eventually become resistant to available therapies, alternative targets like non-coding RNAs, such as microRNAs (miRNAs), are being investigated for their therapeutic potential.

miRNAs are small, non-coding RNAs that bind to mRNA and prevent mRNA translation. One highly conserved family of miRNAs is the miR-200 family [10]. The miR-200 family consists of 5 members, miR-141, miR-200a, miR-200b, miR-200c and miR-429 organized into two clusters (miR-200c/141 and miR-200b/200a/429) [11–13]. The seed sequence, the region of the miRNA predicted to bind to the 3’-UTR of mRNAs, is the same in miR-200b, miR-200c, and miR-429 while miR-200a and miR-141 share the same seed sequence [10]. Therefore, both miR-200 clusters express miRNAs with both miR-200 seed sequences and thus are predicted to share mRNA targets. The best characterized function of the miR-200 family is the regulation of epithelial and mesenchymal phenotypes. miR-200 family members negatively regulate mesenchymal transcription factors like *Zeb1/2*, *Twist1/2*, *Snai1/2*, to maintain epithelial features [13–18]. Therefore, high levels of miR-200s inhibit epithelial mesenchymal transition (EMT) and are associated with reduced migration and invasion of cancer cells [19].

The role of the miR-200 family in ovarian cancer is unclear as conflicting results have been reported. Elevated expression of miR-200s have been associated late stage, high grade disease, poor prognosis or increased tumor growth and metastasis [20–29], however, studies have also associated a decrease in miR-200 expression with late stage disease, poor prognosis or decreased tumor growth and metastasis [30–38]. Of the studies that have manipulated miR-200 expression in ovarian cancer cells, three studies found that miR-200 overexpression inhibited ovarian cancer proliferation and migration. In these studies, transfection of mimics of miR-200b and miR-200c [39] or the miR-200b/200a/429 cluster [39, 40] in ovarian cancer cells inhibited proliferation and migration in vitro. However, studies have also associated elevated miR-200 levels with increased proliferation and migration. Guan et al [29] found that overexpression of miR-200b/200a/429 in the non-transformed ovarian epithelial cell line, T80, stimulated proliferation and promoted growth in soft agar while Suo et al [41] showed that overexpression of miR-200a in OVCAR3 and A2780 ovarian cancer cells increased migration It should be noted that several of these studies only regulated the expression of one miR-200 family member. As mentioned above, miR-200s are expressed as clusters in cells with each cluster expressing at least two miR-200 family members and combined, these two members contain both miR-200 seed sequences. Therefore, studies manipulating miR-200s should alter the expression of entire miR-200 clusters, or both miR-200 clusters, to better represent the physiologic expression of miR-200s in cells.

In this study, we have manipulated both miR-200 clusters in two murine ovarian cancer cell lines (ID8 and 28–2). We found that overexpression of miR-200s reduced the expression of several mesenchymal genes, significantly inhibited proliferation and invasion while increasing apoptosis. Pathway analysis of differentially expressed genes in control and miR-200 overexpressing murine ovarian cancer cells indicated that miR-200s influenced EMT and cell migration. This study represents the first publication of miR-200 manipulation in murine ovarian cancer cells and our findings support the previously published studies that suggest miR-200s inhibit human ovarian cancer progression.

## Materials and methods

### Cell lines

ID8 and 28–2 cells have previously been described [42]. All cells were maintained in DMEM media (GIBCO, Burlington, ON) supplemented with 10% FBS, 2% glutamine, 1% sodium pyruvate, 1% 4-(2-hydroxyethyl)-1-piper- azineethanesulfonic acid (HEPES), and 1% antibiotic/antimycotic. To generate empty vector control cells (ID8EV and 28-2EV), ID8 or 28–2 cells were infected with lentiviral particles containing the pLV[Exp]-EGFP:T2A:Puro-EF1A>mCherry (vector ID: VB160109-10005, VectorBuilder Inc., Chicago, IL, USA). ID8EV and 28-2EV cells were maintained in media supplemented with 30μg/ml puromycin (InvivoGen, San Diego, CA). ID8c141 and 28-2c141 cells were created by infecting ID8 or 28–2 cells with lentiviral particles containing pLV[Exp]-mCherry:T2A:Hygro-CMV>[Ex_miR200c-141]. The miR-200c/141 cluster was synthesized by VectorBuilder and incorporated into the lentiviral vector. ID8c141 cells and 28-2c141 cells maintained in media containing 100μg/ml and 600μg/ml hygromycin (InvivoGen, San Diego, CA) respectively. Lentiviral particles containing pLV[Exp]-GFP:T2A:Puro-CMV>[Ex_miR200b-200a-429] were created from the pLenti 4.1 Ex_miR200b-200a-429 plasmid [43] obtained from Addgene (cat #35533;) and was packaged into a lentiviral vector by VectorBuilder (Chicago, IL). ID8 and 28–2 cells were infected with this vector to create ID8ba429 and 28-2ba429 cells and these cells were maintained in media containing 30μg/ml or 60μg/ml of puromycin, respectively. ID8-200f and 28-2-200f cells were created by infecting ID8 or 28–2 cells with lentiviral particles containing pLV[Exp]-mCherry:T2A:Hygro-CMV>[Ex_miR200c-141] and lentiviral particles containing pLV[Exp]-GFP:T2A:Puro-CMV>[Ex_miR200b-200a-429]. ID8-200f cells were maintained in media containing 40μg/ml puromycin + 50μg/ml hygromycin while 28-2-200f cells were maintained in media containing 60μg/ml puromycin + 1000μg/ml hygromycin.

### RNA extraction, taqman qRT-PCR for microRNA expression and qRT-PCR for gene expression

RNA extraction was perform using a mirVana miRNA Isolation kit (ThermoFisher Scientific, Burlington, ON). For the analysis of miRNA expression, the TaqMan microRNA reverse transcription kit (ThermoFisher Scientific, Burlington, ON) was used to reverse transcript 100 ng of total RNA into cDNA following the manufacturer’s protocol. Detailed description of these protocols have been previously described in [44]. All miR-200 Taqman probes were obtained from ThermoFisher Scientific (Waltham, MA). miR-200 expression was normalized to sno202 and sno234. All gene primers were obtained from Bio-Rad Laboratories (Mississauga, ON), *Cdh1* (qMmuCID0005843), *Hprt*(qMmuCED0045738), *Snai1* (qMmuCID0024342), *Snai2* (qMmuCED0046072), *Twist1*(qMmuCED0004065), *Twist2* (qMmuCID0009652) *Vim* (qMmuCID0005527), *Zeb1* (qMmuCID0009095), and *Zeb2* (qMmuCID0014662). *Hprt* was used as the housekeeping gene.

### Protein isolation and western blotting

Proteins were isolated and western blotting performed as previously described [45]. Primary antibody for Zeb1(cat#70512) was obtained from New England Biolabs Ltd., Whitby, ON, CAN) and was used at a 1:1,000 dilution. Protein bands were visualized using Western ECL substrate (Bio-Rad CAN, Mississauga, ON, CAN) and imaged using the ChemiDocTMXRS+ System (Bio-Rad CAN, Mississauga, ON, CAN). Blots were subsequently normalized with an HRP-linked anti-rabbit β-actin antibody (cat# 5125S; New England Biolabs Ltd., Whitby, ON, CAN) and quantified through the Image Lab software (Bio-Rad CAN, Mississauga, ON, CAN).

### Flow cytometry

BrdU and Annexin V flow cytometry were performed as previously described [46]. Briefly, an APC BrdU flow kit (BD Biosciences, San Jose, CA, cat #552,598) was used following the manufacturer’s protocol for proliferation while the BD Pharminogen APC Annexin V kit (BD Biosciences, San Jose, CA, USA, cat #550475) was used for apoptosis. Analysis was performed using an Accuri C6 cytometer (BD Biosciences, San Jose, CA) using a flow rate of 35 μl/min and 25,000 events were collected.

### Transwell invasion assay

Transwell invasion assays were performed using Matrigel coated inserts from the BioCoat Matrigel Invasion Chamber kit (Corning, NY) were used as previously described [47]. 75,000 cells for each cell line were seeded in the upper chamber in serum-free media and the lower chamber was filled with media containing 10% FBS. Invasion was assessed after 24 hours.

### RNA sequencing

RNA sequencing was performed by Novogene Corporation Inc. (Sacramento, CA, USA) using total RNA extracted with the miRVana miRNA isolation kit (ThermoFisher Scientific, Burlington, ON, Canada) as previously described [48]. Three independent samples were sequenced for cell line. Fastq files were processed using Genialis software v3.0 (Genialis Inc, Houston, TX, USA) following the standard RNA-seq pipeline which uses BBDuk to remove adapters and trim reads, STAR to align the reads, and featureCounts to generate gene-level counts. Heatmap analysis was performed using Genialis software v3.0 (Genialis Inc., Houston, TX, USA), and pathway analysis was performed using Enrichr software v3.0 [49, 50]. The RNA sequencing data have been uploaded to the GEO database as accession number GSE250195.

### Statistical analysis

Statistical analysis for miR-200 expression was determined using an ANOVA followed by a Dunnett’s test while all other comparisons used an unpaired t-test using GraphPad Prism 10 (GraphPad Software, San Diego, CA, USA). Values were considered statistically significant when p < 0.05.

## Results

### Overexpression of miR-200s in ID8 and 28–2 cells

**Fig 1:**
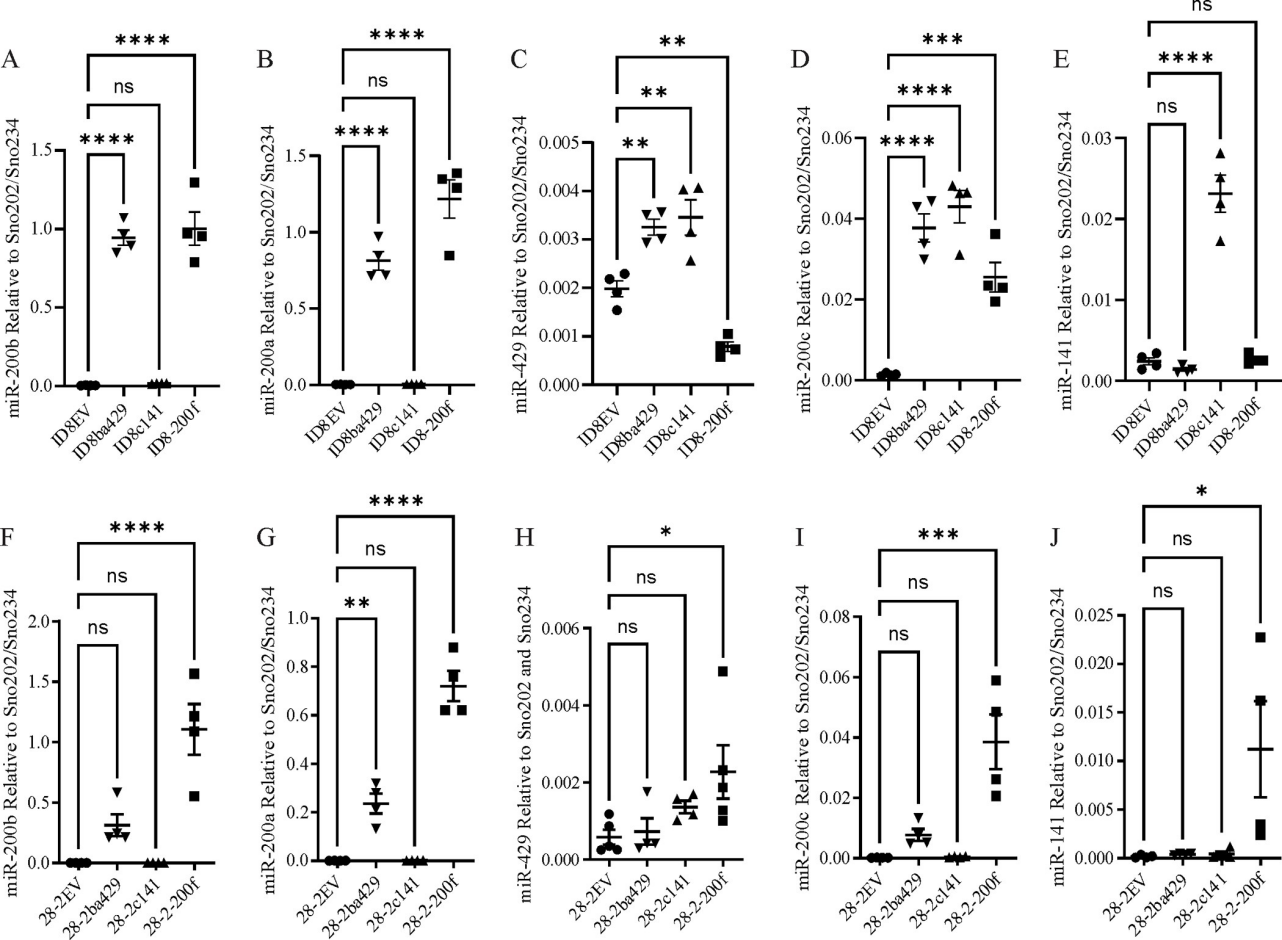
Taqman RT-PCR for miR-200 family members in (A-E) ID8 or (F-J) 28–2 cells infected with an empty vector (EV) lentivirus, a lentivirus expressing the miR-200b/200a/429 cluster (ba429), a lentivirus expressing the miR-200c/141 cluster (c141) or both clusters (200f). miR-200 expression was normalized to Sno202 and Sno234 and is presented as the mean (horizonal line) and standard error (error bars). Four biological replicates were used for each cell line. *p < 0.05, **p < 0.01, ***p < 0.001, ****p < 0.0001.

To evaluate the impact of miR-200s in ovarian cancer, murine ID8 and 28–2 cells were infected with lentiviral constructs containing an empty vector (EV), the miR-200b/200a/429 cluster (ba429), the miR-200c/141 cluster (c141) or both clusters (200f). As shown in Fig 1 re-expression of the ba429 cluster significantly increased the expression of miR-200b (Fig 1A), miR-200a (Fig 1B), and miR-429 (Fig 1C) in ID8 cells. Interestingly, re-expression of the miR-200b/200a/429 cluster also significantly increased the expression of miR-200c (Fig 1D) but not miR-141 (Fig 1E). Re-expression of the miR-200c/141 cluster in ID8 cells significantly increased the expression of miR-200c (Fig 1D) and miR-141 (Fig 1E). Our attempt to overexpress both clusters in the ID8 cells led to a significant increase in miR-200b (Fig 1A), miR-200a (Fig 1B), and miR-200c (Fig 1D) but not miR-429 (Fig 1C) or miR-141 (Fig 1E).

For the 28–2 cells, expression of the miR-200b/200a/429 cluster resulted in a significant increase in miR-200a (Fig 1G) but not miR-200b (Fig 1F) or miR-429 (Fig 1H) while expression of the miR-200c/141 cluster did not significantly increase the expression of miR-200c (Fig 1I) or miR-141 (Fig 1J). Expression of both clusters lead to a significant increase in all five miR-200 family members (Fig 1F–1H). It should be noted that miR-429 and miR-141 are expressed at considerably lower levels than miR-200a, miR-200b and miR-200c in both cell lines. Therefore, miR-200a, miR-200b and miR-200c are likely the main miR-200s driving the effects observed in ID8-200f and 28-2-200f cells.

### The impact of miR-200 overexpression on epithelial and mesenchymal gene expression

**Fig 2:**
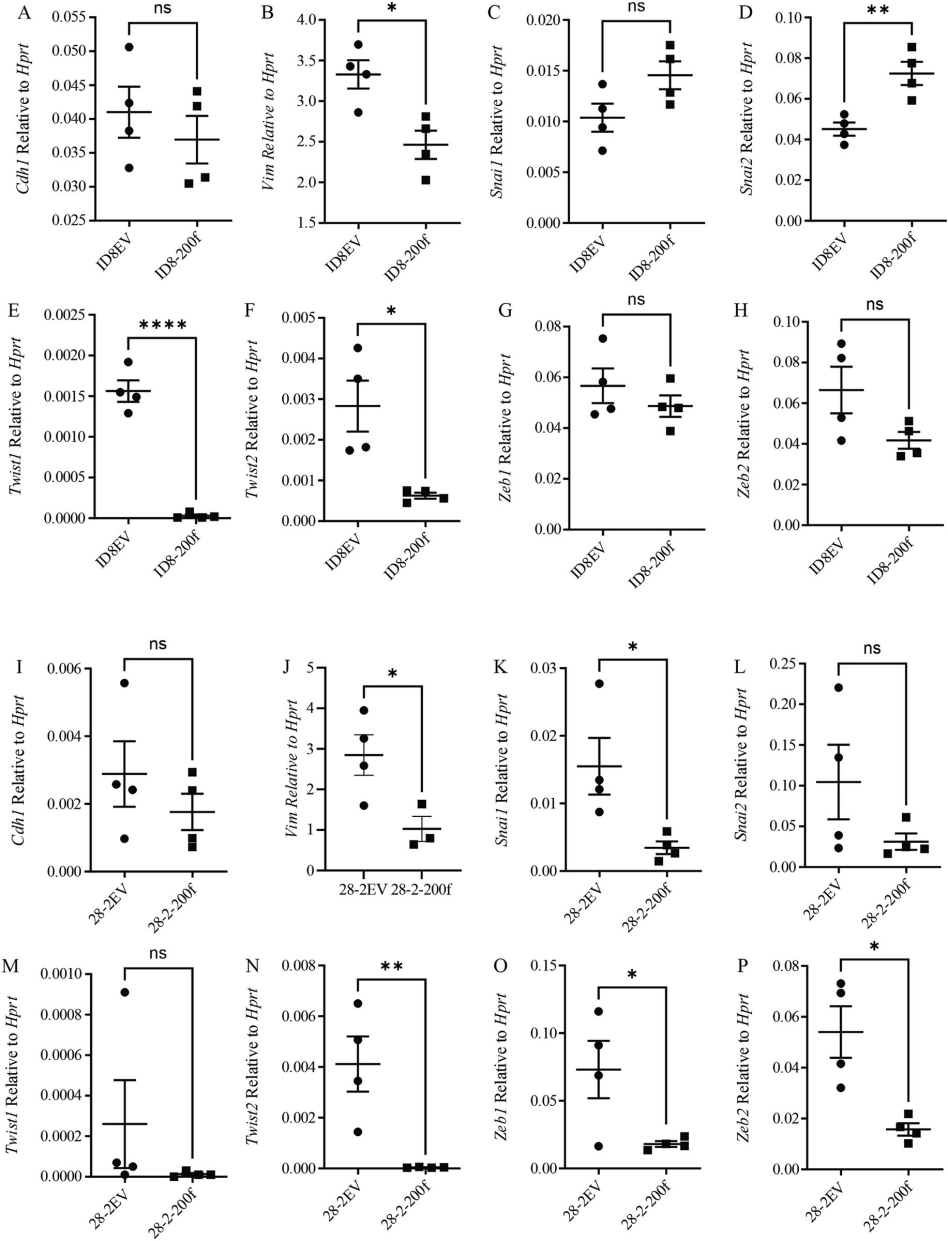
Quantitative RT-PCR for (A,I) *Cdh1*, (B,J) *Vim*, (C,K) *Snai1*, (D,L) *Snai2*, (E,M) *Twist1*, (F,N) *Twist2*, (G,O) *Zeb1*, and (H,P) *Zeb2* in (A-H) ID8EV and ID8-200f cells or (I-P) 28-2EV and 28-2-200f cells. All genes were normalized to HPRT and the horizontal lines represent the means and the error bars represent the standard error. Four biological replicates were used for each cell line. *p < 0.05, **p < 0.01, ****p < 0.0001.

For all subsequent experiments, we focused on the empty vector samples (ID8EV and 28-2EV) compared to the cell line expressing both miR-200 clusters (ID8-200f and 28-2-200f). In the ID8-200f cells, there was a significant reduction in *Vim* (Fig 2B), *Twist1* (Fig 2E), and *Twist2* (Fig 1F) with no significant change in *Cdh1* (Fig 2A), *Snai1*, (Fig 2C) *Zeb1* (Fig 2G), *Zeb2* (Fig 2H) compared to ID8EV cells. There was also a significant increase in *Snai2* (Fig 2D) in the ID8-200f cells compared to ID8EV cells. For the 28-2-200f cells, there was a significant reduction in *Vim* (Fig 2J), *Snai1* (Fig 2K), *Twist2* (Fig 2N), *Zeb1* (Fig 2O), and *Zeb2* (Fig 2P) compared to 28-2EV cells. There were no significant changes in *Cdh1* (Fig 2I), *Snai2* (Fig 2L), or *Twist1* (Fig 2M) between 28-2EV and 28-200f cells.

**Fig 3:**
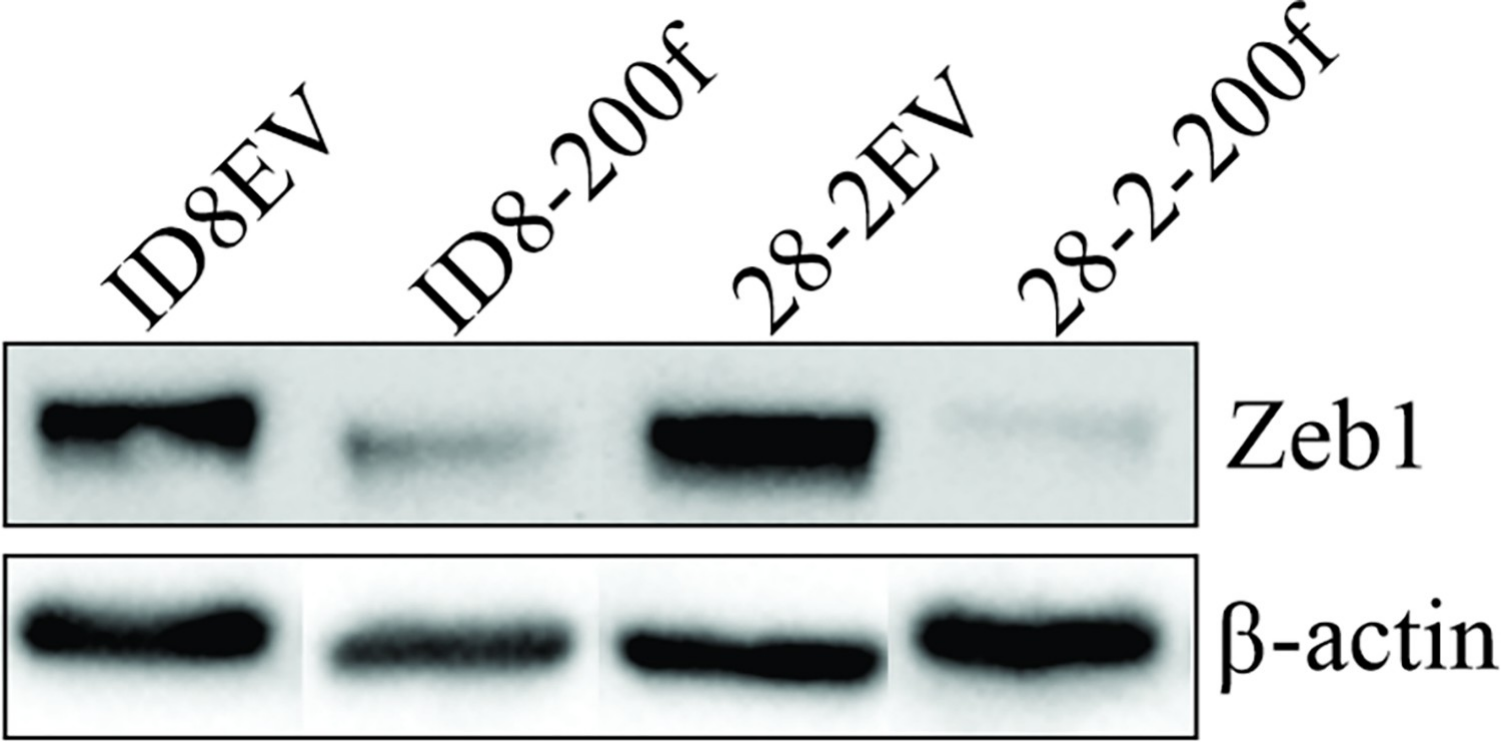
Representative western blot of Zeb1 in ID8EV, ID8-200f, 28-2EV and 28-2-200f cells. β-actin was used as a loading control. This western was repeated using a second set of samples to confirm the results.

Since miR-200s may impair mRNA translation rather than mRNA expression, a known target of miR-200s, Zeb1, was evaluated at the protein level to confirm we have altered miR-200 function. As shown in Fig 3, Zeb1 protein levels were reduced in ID8-200f and 28-2-200f cells compared to their respective controls confirming that elevated levels of miR-200 functionally reduced the levels of mesenchymal proteins.

### Overexpression of miR-200s reduce proliferation and invasion while increasing apoptosis

**Fig 4:**
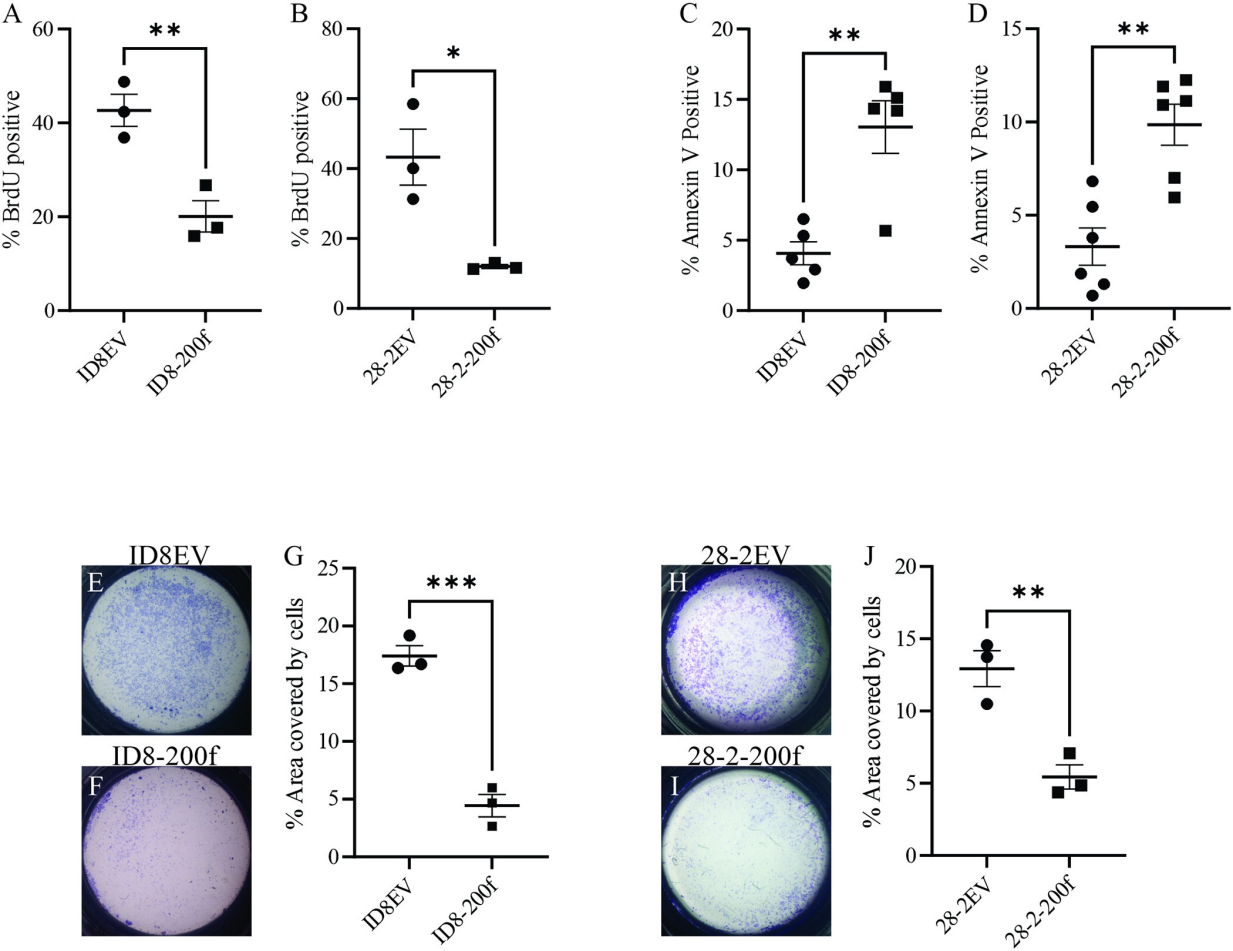
Percent BrdU positive cells for (A) ID8EV and ID8-200f and (B) 28-2EV and 28-2-200f cells (B) as determined by flow cytometry. Percent annexin V positive cells for (C) ID8EV and ID8-200f and (D) 28-2EV and 28-2-200f cells as determined by flow cytometry. Representative transwell images for (E) ID8EV, (F) ID8-200f, (H) 28-2EV, and (I) 28-2-200f cells. Quantification of the average percent of the transwell insert covered by invaded cells for (G) ID8EV and ID8-200f and (J) 28-2EV and 28-2-200f cells. Three biological replicates were used for BrdU and the invasion assay while five or six biological replicates were used for annexin V. *p < 0.05, **p < 0.01, ***p < 0.001.

Proliferation and apoptosis were assessed using BrdU and Annexin V flow cytometry, respectively. Proliferation was significantly reduced more that 2-fold in both ID8-200f and 28-2-200f cells compared to their respective controls (Fig 4A–4D)). Along with the reduced proliferation, basal apoptotic rates were significantly increased approximately 3-fold in both ID8-200f and 28-2-200f cells compared to their respective controls. Cell invasion was assessed using a Matrigel coated transwell assay. As shown in Fig 4E–4J, overexpression of miR-200s in ID8 and 28–2 cells significantly reduced invasion in both cell lines.

### Overexpression of miR-200s regulate the expression of genes related to EMT and cell migration

RNA sequencing was performed on ID8EV, ID8-200f, 28-2EV and 28-2-200f cells. Using a cut-off of logFC > 1 or logFC <-1 and false discovery rate (FDR) < 0.01, there were 1011 genes differentially expressed between ID8EV and ID8-200f cells. 243 genes were upregulated, and 786 genes were downregulated in ID8-200f cells compared to ID8EV cells. The top 10 differentially expressed genes based on FDR are listed in S1 Table. Using Enrichr to analyze these 1011 differentially expressed genes, the top ENCODE and ChEA Consensus TFs from ChIP-X was SUZ12 and the top MSigDB Hallmark 2020 was Epithelial Mesenchymal Transition (S2 Table). The top ontologies were Regulation of Cell Migration (GO Biological Process 2023), Collagen-Containing Extracellular Matrix (GO Cellular Component 2023), and Transmembrane Receptor Protein Kinase Activity (GO Molecular Function 2023) (S2 Table).

RNA sequencing was also performed to identify genes altered by the miR-200 family in 28–2 cells. Using a cut-off of logFC > 1 or logFC <-1 and false discovery rate (FDR) < 0.01, there were 753 genes differentially expressed between 28-2EV and 28-2-200f cells. 377 genes were upregulated, and 376 genes were downregulated in 28-2-200f cells compared to 28-2EV cells. The top 10 differentially expressed genes based on FDR are listed in S3 Table. Using Enrichr to analyze these 753 differentially expressed genes, the top ENCODE and ChEA Consensus TFs from ChIP-X was SUZ12, the top MSigDB Hallmark 2020 was Epithelial Mesenchymal Transition (S4 Table). The top ontologies were Regulation of Cell Migration (GO Biological Process 2023), Collagen-Containing Extracellular Matrix (GO Cellular Component 2023), and GTPase Regulator Activity (GO Molecular Function 2023) (S4 Table).

**Fig 5:**
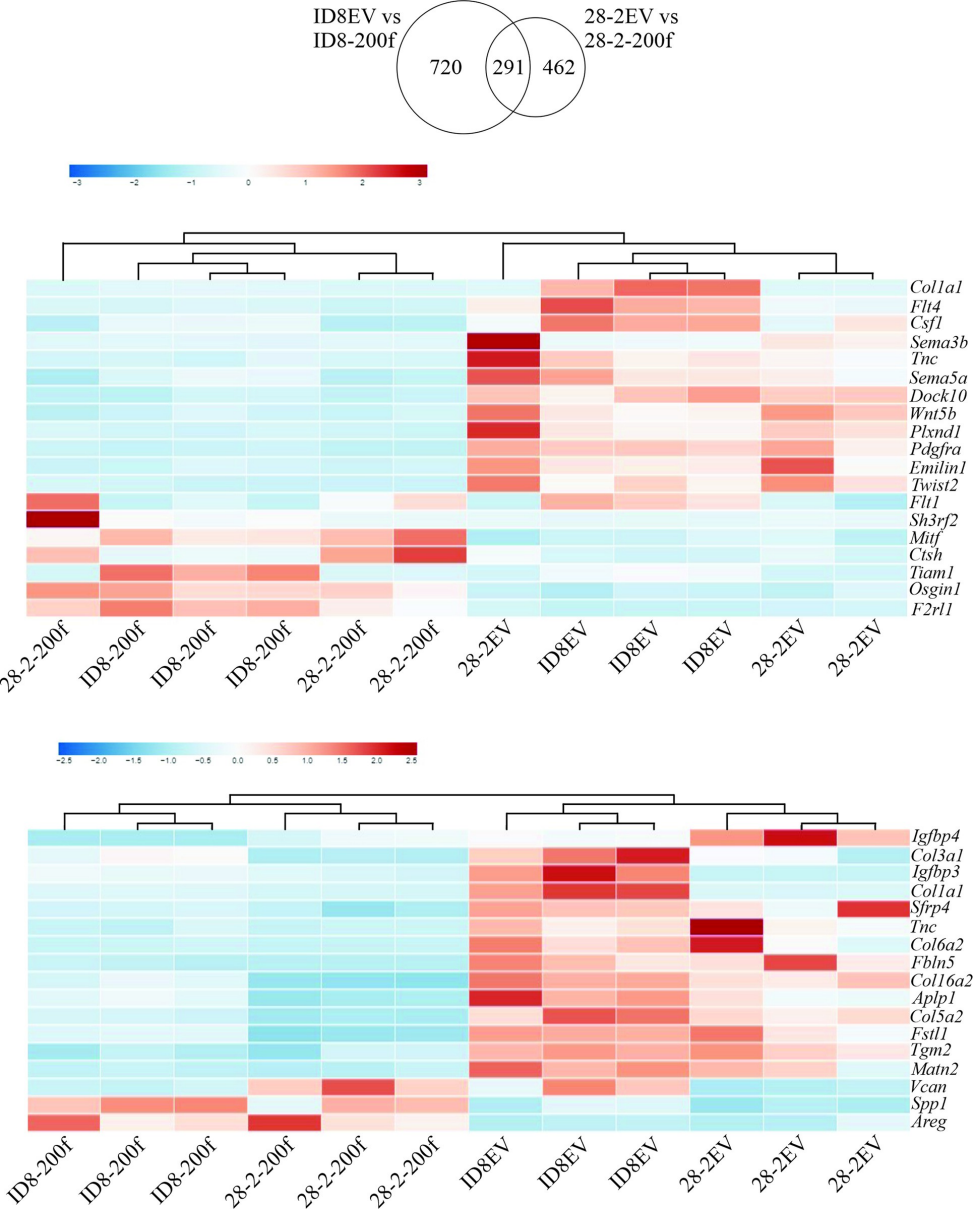
(A) Venn diagram showing the number of unique and shared genes as determined by RNA sequencing in ID8EV vs ID8-200f and 28-2EV vs 28-2-200f. Heatmap of (B) shared genes implicated in EMT and (C) shared genes implicated in cell migration. Three biological replicates were sequenced for each cell line.

Of the 1011 differentially expressed genes in the ID8EV vs ID8-200f comparison and 753 genes in the 28-2EV vs 28-2-200f comparison, 291 genes were shared (Fig 5A). Analyzing the shared genes with Enrichr, the top ENCODE and ChEA Consensus TFs from ChIP-X was SUZ12, the top MSigDB Hallmark 2020 was Epithelial Mesenchymal Transition (S5 Table). The top ontologies were Regulation of Cell Migration (GO Biological Process 2023), Endoplasmic Reticulum Lumen (GO Cellular Component 2023), and Glutathione Transferase Activity (GO Molecular Function 2023) (S5 Table).

Since miR-200s are known to regulate EMT and the top MSigDB Hallmark was EMT, the shared genes were analyzed for genes that were attributed to EMT. Nineteen shared genes were found that regulate EMT and these genes were *Sh3rf2*, *Pdgfra*, *Ctsh*, *Flt1*, *Csf1*, *Twist2*, *Col1a1*, *Sema5a*, *Sema3b*, *F2rl1*, *Flt4*, *Tiam1*, *Tnc*, *Dock10*, *Plxnd1*, *Emilin1*, *Mitf*, *Osgin1*, and *Wnt5b* (Fig 5B). Furthermore, as our results showed that miR-200s significantly reduced invasion in both cell lines, the shared genes were analyzed for genes that were attributed to cell migration. Seventeen genes were found that regulate migration and these genes were *Aplp1*, *Areg*, *Col16a1*, *Col1a1*, *Col3a1*, *Col5a1*, *Col6a2*, *Fbln5*, *Fstl1*, *Igfbp3*, *Igfbp4*, *Matn2*, *Sfrp4*, *Spp1*, *Tgm2*, *Tnc*, and *Vcan* (Fig 5C).

Of the 36 shared genes that contribute to EMT and migration, 20 of the genes were determined to be direct targets of members of the miR-200 family, using the miRNA prediction software miRWalk [51]. These genes were *Aplp1*, *Col16a1*, *Col1a1*, *Col3a1*, *Csf1*, *Ctsh*, *Dock10*, *Emilin1*, *Flt4*, *Fstl1*, *Igbfbp3*, *Mitf*, Pdgfra, *Sema3b*, *Sema5a*, *Sh3rf2*, *Tgm2*, *Tiam1*, *Tnc*, and *Vcan*. Additionally, since the top ENCODE and ChEA Consensus TF from ChIP-X was SUZ12, Harmonizome 3.0 (Rouillard et al., 2016) was used to detect genes that were targets of SUZ12. Of the 291 shared genes from ID8EV vs ID8-200f and 28-2EV vs 28-2-200f, 122 of these shared genes (~42%) were targets of SUZ12. In addition, 20 of the 36 genes (~56%) related to EMT and migration were found to be target genes of SUZ12. These genes were *Areg*, *Col16a1*, *Col5a1*, *Fbln5*, *Fstl1*, *Igfbp3*, *Igfbp4*, *Vcan*, *Pdgfra*, *Ctsh*, *Flt1*, *Csf1*, *Twist2*, *Sema5a*, *Sema3b*, *Flt4*, *Flt1*, *Dock10*, *Plxnd1*, and *Wnt5b*.

## Discussion

In this study, we successfully increased the expression of both miR-200 seed sequences in two murine ovarian cancer cell lines, ID8 and 28–2. There were a few surprising findings including the significant increase in miR-429 in the ID8c141 cells and the significant increase in miR-200c in ID8ba429 cells. We have also observed this phenomenon in the human breast cancer cell line MDA-MB-231 where overexpression of the miR-200c/141 cluster induced a significant increase in miR-200b [52]. It remains unclear whether miR-200 clusters can, under some circumstances, regulate each other’s expression or whether certain conditions allow primers specific for one miR-200 family member to bind to and amplify a different miR-200 family member as the sequences of some of the miR-200s are highly similar. For example, the sequence for mmu-miR-200b-3p is UAAUACUGCCUGGUAAUGAUGA while the sequence for mmu-miR-200c-3p is UAAUACUGCCGGGUAAUGAUGGA.

Overexpression of miR-200s in ID8 and 28–2 cells significantly reduced proliferation and invasion while increasing apoptosis in both cell lines. These findings are consistent with several previous papers that showed increasing one or more miR-200 family members in human ovarian cancer cell lines could inhibit proliferation [39, 53, 54], or migration/invasion [39, 54–59] and increase apoptosis [53]. However, several studies found that miR-200s had the opposite effect to those observed in this study. Guan et al [29] transiently transfected miR-200a, miR-200b and miR-429 mimics in the non-tumorigenic human ovarian epithelial cell line T80 and showed that increasing miR-200 levels promoted proliferation, soft agar growth and xenograft tumor formation. In another study, transfection of ES2 or SKOV3 cells with a miR-200a-3p mimic was shown to increase proliferation, colony formation and invasion [60]. Liu et al [61] showed that overexpression of miR-200a in OVCAR3 cells promoted proliferation while Suo et al [41] found that overexpression of a miR-200a mimic increased, while a miR-200a inhibitor decreased, invasion of OVCAR3 cells. Finally, in a study by Ibrahim et al [62], it was found that a miR-200c mimic increased proliferation but decreased invasion of CAOV3 and SKOV3 cells.

The reason behind these divergent results remains unclear but one variable is that most of the studies only altered the expression of an individual miR-200 and in some cases, only the 3p or 5p sequence of an individual miR-200. While the regulation of individual miR-200s is useful for identifying mRNAs targeted by specific miR-200s, the physiological relevance is less clear. Since miR-200s are expressed as clusters in cells, at a minimum a whole miR-200 cluster should be altered to represent a physiologically relevant change in miR-200 expression. Expressing an individual cluster or both clusters ensure that both miR-200 seed sequences are expressed.

Only one published study used lentiviral vectors to stably elevate the expression of the miR-200b/200a/429 cluster, the miR-200c/141 cluster or both clusters and this was performed in a spontaneously immortalized human ovarian surface epithelial cell line OSE7. Overexpression of the individual clusters or co-overexpression of both clusters in OSE7 cells significantly reduced proliferation [63]. Apoptosis and invasion were not evaluated in this study.

Of the studies that evaluated the expression of epithelial and mesenchymal genes, elevated expression of miR-200s increased the expression of epithelial genes like *Cdh1* and decreased the expression of mesenchymal genes such as *Zeb1* and *Zeb2* [54, 57–59, 64–67] and thus were consistent with our findings. The maintenance of epithelial identity by miR-200s is one of the best characterized functions of the miR-200 family [14–16]. Since EMT is thought to enhance metastasis, it is somewhat surprising that miR-200s were consistently observed to downregulate mesenchymal genes or upregulate epithelial genes in ovarian cancer cells yet some studies found that elevated levels of miR-200s promoted migration or invasion [41, 60]. It should be noted that only four papers were found that evaluated both EMT gene expression and migration or invasion [54, 57, 58, 63]. In all four of these publications, miR-200 overexpression decreased mesenchymal gene expression and/or increased epithelial gene expression and inhibited migration and/or invasion. The role of miR-200s in EMT was also supported by our RNA-sequencing. Pathway analysis of genes altered by miR-200s identified EMT as a pathway affected in both ID8-200f and 28-2-200f compared to their respective control lines. Two previous studies performed microarrays following miR-200 overexpression in human ovarian cells and these studies also found EMT to be one of the top regulated pathways [63, 65]. Therefore, most of studies on human ovarian cell lines agree with our findings in murine ovarian cancer cells that miR-200s reduce mesenchymal gene expression and invasion.

Another interesting finding in the pathway analysis was that the top ENCODE and ChEA Consensus TF from ChIP-X was SUZ12. This is consistent with our studies that overexpressed miR-200s in murine and human mammary tumor cell lines [48]. Suppressor of zeste 12 (SUZ12) is a key component of the polycomb repressive complex 2 (PRC2). The PRC2 is responsible for mono, di- and tri-methylating histone H3 on lysine 27 (H3K27). Methylation of H3K27 is associated with repression of gene expression [68]. Exactly how miR-200s regulate PRC2 activity remains to be determined but this pathway would provide an additional mechanism that miR-200s could employ to regulate gene expression.

## Conclusion

This is the first study to stably overexpress miR-200s in murine ovarian cancer cells and show that miR-200s decrease mesenchymal gene expression, inhibit proliferation and invasion and increase apoptosis. These cells should be useful for furthering our understanding of miR-200s in human ovarian cancer since miR-200s have been shown to suppress mesenchymal gene expression and reduce migration and invasion in human ovarian cancer. In addition, the mature sequences of miR-141, miR-200a, miR-200b and miR-200c are identical in mice and humans and thus should target similar genes in murine and human cells. Moreover, we have previously shown that injection of ID-8 or 28–2 cells into the ovaries of syngeneic C57Bl/6 mice replicate human ovarian cancer progression including ascites formation and metastasis [42]. Therefore, the cells characterized in this study can be used to model the impact of elevated miR-200 levels on ovarian cancer progression in an in vivo model with an intact immune system. These future studies will determine whether miR-200s impair primary tumor growth and or metastasis in vivo and thus represent a potential therapeutic strategy for ovarian cancer.

## Supporting information

S1 Table Top 10 differentially expressed genes, based on false discovery rate (FDR) in ID8-200f cells compared to ID8EV cells. Genes with negative log fold change are downregulated in ID8-200f cells compared to ID8EV cells.
(PDF)

S2 Table Top transcription factors and pathways identified by Enrichr from the differentially expressed genes in ID8-200f cells compared to ID8EV cells. (PDF)

S3 Table Top 10 differentially expressed genes, based on false discovery rate (FDR) in 28-2-200f cells compared to 28-2EV cells. Genes with negative log fold change are downregulated in 28-2-200f cells compared to 28-2EV cells.
(PDF)

S4 Table Top transcription factors and pathways identified by Enrichr from the differentially expressed genes in 28-2-200f cells compared to 28-2EV cells. (PDF)

S5 Table Top transcription factors and pathways identified by Enrichr from the shared differentially expressed genes in the ID8-200f cells compared ID8EV cells and 28-2-200f cells compared to 28-2EV cells. (PDF)

S1 Raw image Western blot showing the entire gel for (A) Zeb1 and (B) Hprt. Fig 3 was adapted from this western blot.
(TIF)

## Abbreviations

ba429: miR-200b/200a/429 cluster
BrdU: bromodeoxyuridine
c141: miR-200c/141 cluster
CCC: clear cell carcinoma
EC: endometrioid carcinoma
EMT: epithelial mesenchymal transition
EV: empty vector
FDR: false discovery rate
HGSOC: high grade serous ovarian cancer
LGSOC: low grade serous ovarian cancer
MC: mucinous carcinoma
miRNA: microRNA
PARP: poly (ADP-ribose) polymerase
PRC2: polycomb repressor complex 2
UTR: untranslated region
