# Supplementary material for: Overexpression of miR-200s inhibits proliferation and invasion while increasing apoptosis in murine ovarian cancer cells
Source: PLoS One. 2024 Jul 19;19(7):e0307178. doi: 10.1371/journal.pone.0307178 (PMC11259287; doi:10.1371/journal.pone.0307178)
Supplement: S1 Table — Genes with negative log fold change are downregulated in ID8-200f cells compared to ID8EV cells. (PDF) [file pone.0307178.s001.pdf]

S1 Table: Top 10 genes differentially expressed in ID8-200f cells compared to ID8EV cells

Gene ID	Gene Symbol	Log Fold Change[1]	FDR
ENSMUSG00000023039	*Krt7*	5.5	$2.1x10^{-243}$
ENSMUSG00000017493	*Igfbp4*	-2.7	$1.8x10^{-186}$
ENSMUSG00000001506	*Col1a1*	-3.6	$4.6x10^{-146}$
ENSMUSG00000074625	*Arhgap40*	3.8	$3.4x10^{-133}$
ENSMUSG00000025608	*Podxl*	-4.3	$7.0x10^{-119}$
ENSMUSG00000022197	*Pdzd2*	-6.5	$2.3x10^{-93}$
ENSMUSG00000023092	*Fhl1*	-2.7	$1.3x10^{-89}$
ENSMUSG00000034981	*Parm1*	2.3	$1.3x10^{-86}$
ENSMUSG00000026726	*Cubn*	-4.5	$1.6x10^{-86}$
ENSMUSG00000054889	*Dsp*	2.6	$2.0x10^{-86}$

[1]Genes downregulated in ID8200f cells relative to ID8EV cells show negative log fold changes.
