# Supplementary material for: Overexpression of miR-200s inhibits proliferation and invasion while increasing apoptosis in murine ovarian cancer cells
Source: PLoS One. 2024 Jul 19;19(7):e0307178. doi: 10.1371/journal.pone.0307178 (PMC11259287; doi:10.1371/journal.pone.0307178)
Supplement: S2 Table — (PDF) [file pone.0307178.s002.pdf]

S2 Table: Transcription (a) and Pathways (b) analysis of genes differentially regulated between ID8EV and ID8-200f cells

ENCODE and ChEA Consensus TFs from Chip-X	Adjusted p-value
SUZ12 CHEA	1.3×10^{-16}
SMAD4 CHEA	1.4×10^{-3}
MSigDB Hallmark 2020	
Epithelial Mesenchymal Transition	1.1×10^{-16}
TNF-alpha Signaling via NF-kB	5.1×10^{-6}
GO Biological Process 2023	
Regulation Of Cell Migration	1.3×10^{-4}
Extracellular Structure Organization	1.3×10^{-4}
GO Cellular Component 2023	
Collagen-Containing Extracellular Matrix	6.8×10^{-10}
Endoplasmic Reticulum Lumen	5.9×10^{-3}
GO Molecular Function 2023	
Transmembrane Receptor Protein Kinase Activity	7.4×10^{-4}
Semaphorin Receptor Binding	2.0×10^{-3}
