# Supplementary material for: Overexpression of miR-200s inhibits proliferation and invasion while increasing apoptosis in murine ovarian cancer cells
Source: PLoS One. 2024 Jul 19;19(7):e0307178. doi: 10.1371/journal.pone.0307178 (PMC11259287; doi:10.1371/journal.pone.0307178)
Supplement: S3 Table — Genes with negative log fold change are downregulated in 28-2-200f cells compared to 28-2EV cells. (PDF) [file pone.0307178.s003.pdf]

S3 Table: Top 10 genes differentially expressed in 28-2-200f cells compared to 28-2EV cells

Gene ID	Gene Symbol	Log Fold Change[1]	FDR
ENSMUSG00000017607	*Tns4*	5.1	5.8E-135
ENSMUSG00000074625	*Arhgap40*	4.6	1.2E-69
ENSMUSG00000058254	*Tspan7*	6.4	9.5E-69
ENSMUSG00000038677	*Scube3*	-8.9	3.5E-59
ENSMUSG00000028782	*Adgrb2*	-4.0	4.4E-53
ENSMUSG00000078952	*Lncenc1*	5.1	3.6E-40
ENSMUSG00000040690	*Col16a1*	-3.9	3.4E-36
ENSMUSG00000000308	*Ckmt1*	6.3	9.8E-36
ENSMUSG00000019852	*Arfgef3*	5.5	2.1E-27
ENSMUSG00000032380	*Dapk2*	3.8	1.2E-26

[2]Genes downregulated in 28-2-200f cells relative to 28-2EV cells show negative log fold changes.
