# Supplementary material for: Overexpression of miR-200s inhibits proliferation and invasion while increasing apoptosis in murine ovarian cancer cells
Source: PLoS One. 2024 Jul 19;19(7):e0307178. doi: 10.1371/journal.pone.0307178 (PMC11259287; doi:10.1371/journal.pone.0307178)
Supplement: S4 Table — (PDF) [file pone.0307178.s004.pdf]

S4 Table: Transcription (a) and Pathways (b) analysis of genes differentially regulated between 28-2EV and 28-2-200f cells

ENCODE and ChEA Consensus TFs from Chip-X	Adjusted p-value
SUZ12 CHEA	2.4×10^{-9}
NFE2L2 CHEA	1.3×10^{-2}
MSigDB Hallmark 2020	
Epithelial Mesenchymal Transition	7.0×10^{-12}
KRAS Signaling Up	1.4×10^{-5}
GO Biological Process 2023	
Regulation Of Cell Migration	1.6×10^{-9}
Positive Regulation of Cell Migration	1.1×10^{-6}
GO Cellular Component 2023	
Collagen-Containing Extracellular Matrix	1.9×10^{-7}
Endoplasmic Reticulum Lumen	1.3×10^{-5}
GO Molecular Function 2023	
GTPase Regulator Activity	3.8×10^{-4}
Frizzled Binding	6.1×10^{-4}
