# Supplementary material for: Overexpression of miR-200s inhibits proliferation and invasion while increasing apoptosis in murine ovarian cancer cells
Source: PLoS One. 2024 Jul 19;19(7):e0307178. doi: 10.1371/journal.pone.0307178 (PMC11259287; doi:10.1371/journal.pone.0307178)
Supplement: S5 Table — (PDF) [file pone.0307178.s005.pdf]

S5 Table: Transcription (a) and Pathways (b) analysis of genes in common between genes that were differentially regulated for 28-2EV and 28-2-200f cells and genes that were differentially regulated between ID8EV and ID8-200f cells

ENCODE and ChEA Consensus TFs from Chip-X	Adjusted p-value
SUZ12 CHEA	1.5×10^{-5}
NFE2L2 CHEA	4.6×10^{-2}
MSigDB Hallmark 2020	
Epithelial Mesenchymal Transition	3.5×10^{-8}
Angiogenesis	1.8×10^{-3}
GO Biological Process 2023	
Regulation of Cell Migration	1.8×10^{-2}
Positive Regulation of Cell Motility	1.8×10^{-2}
GO Cellular Component 2023	
Endoplasmic Reticulum Lumen	6.3×10^{-6}
Collagen-Containing Extracellular Matrix	5.0×10^{-5}
GO Molecular Function 2023	
Glutathione Transferase Activity	3.3×10^{-5}
Platelet-Derived Growth Factor Binding	1.2×10^{-3}
