# Supplementary figures and images for: Overexpression of miR-200s inhibits proliferation and invasion while increasing apoptosis in murine ovarian cancer cells
Source: PLoS One. 2024 Jul 19;19(7):e0307178. doi: 10.1371/journal.pone.0307178 (PMC11259287; doi:10.1371/journal.pone.0307178)

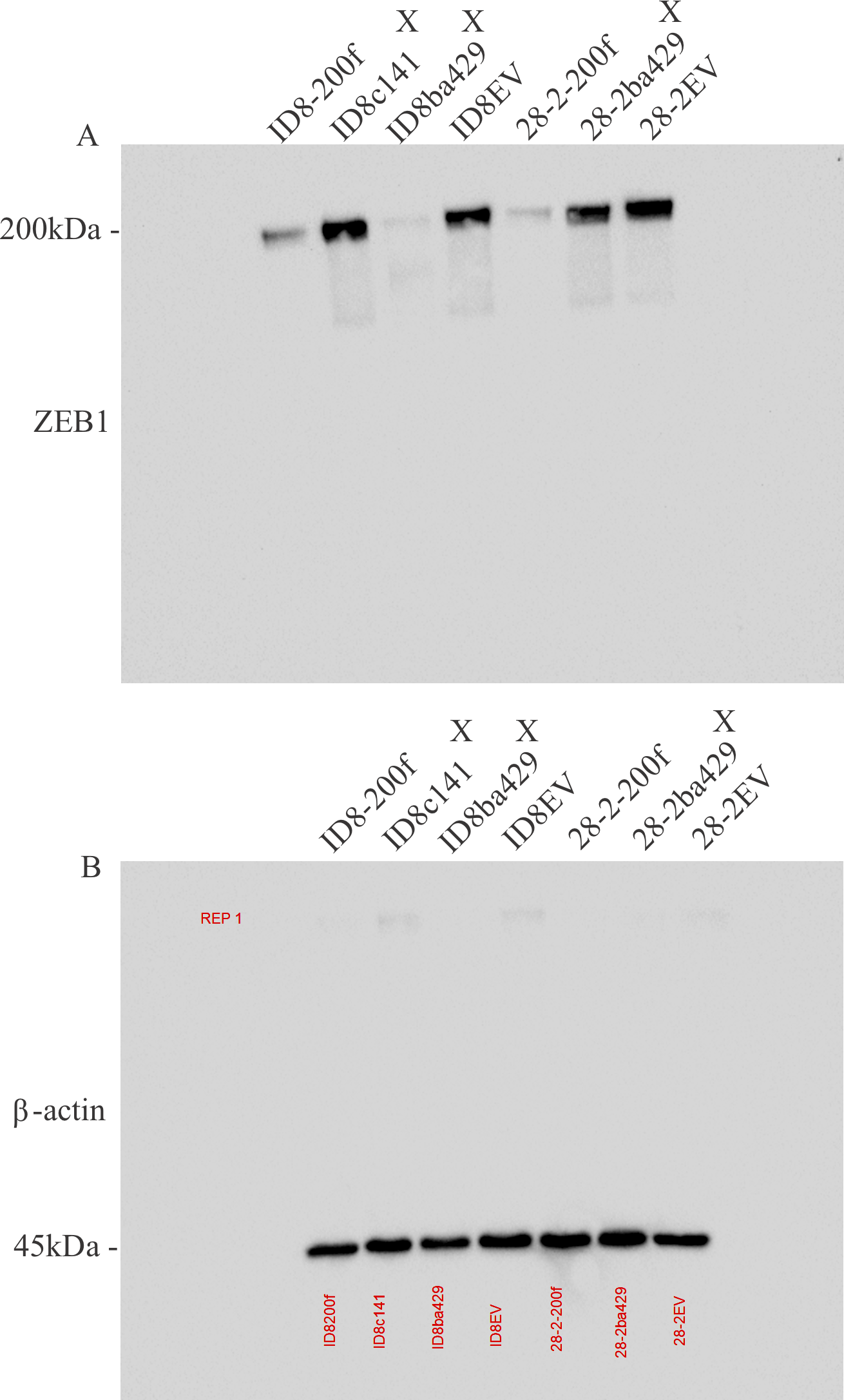

Supplement: S1 Raw image — Western blot showing the entire gel for (A) Zeb1 and (B) Hprt. Fig 3 was adapted from this western blot. (TIF) [file pone.0307178.s006.tif]
